# Independent determinants of prolonged emergency department length of stay in a tertiary care centre: a prospective cohort study

**DOI:** 10.1186/s13049-018-0547-5

**Published:** 2018-09-20

**Authors:** Daniël van der Veen, Claudia Remeijer, Anne J. Fogteloo, Christian Heringhaus, Bas de Groot

**Affiliations:** 1 Department of Emergency Medicine, Leiden University Medical Centre, Albinusdreef 2, 2300 RC Leiden, the Netherlands; 2 Department of Internal Medicine, Leiden University Medical Centre, Albinusdreef 2, 2300 RC Leiden, the Netherlands

**Keywords:** Emergency department, Overcrowding, Emergency department length of stay

## Abstract

**Background:**

Emergency department (ED) overcrowding is a potential threat for patient safety. We searched for independent determinants of prolonged ED length of stay (LOS) with the aim to identify factors which can be targeted to reduce ED LOS, which may help in preventing overcrowding.

**Methods:**

This prospective cohort study included consecutive ED patients in a Dutch tertiary care centre. Multivariable logistic regression analysis was used to identify independent determinants of ED LOS > 4 h, including patient characteristics (demographics, referral type, acuity, (number of) presenting complaints and comorbidity), treating specialty, diagnostic testing, consultations, number of patients in the ED and disposition. Furthermore, we quantified the absolute time delays (measured in real-time) associated with the most important independent determinants of prolonged ED LOS.

**Results:**

In 1434 included patients independent determinants of prolonged ED LOS were number and type of presenting complaints, specialty, laboratory/radiology testing and consultations, and ICU admission. *Modifiable* determinants with the largest impact were blood testing; Adjusted odds ratio (AOR (95%-CI)); 3.45 (1.95–6.11), urine testing; 1.79 (1.21–2.63), radiology imaging; 3.02 (2.13–4.30), and consultation; 5.90 (4.08–8.54).

Combined with the laboratory/radiology testing and/or consultations (requested in 1123 (78%) patients) the decision-making and discharge process consumed between 74 (42%) and 117 (66%) minutes of the total ED LOS of 177 (IQR: 129–225) minutes.

**Conclusions:**

In tertiary care EDs, ED LOS can be reduced if the process of laboratory/radiology testing and consulting is optimized and the decision-making and discharge procedures are accelerated.

**Electronic supplementary material:**

The online version of this article (10.1186/s13049-018-0547-5) contains supplementary material, which is available to authorized users.

## Background

### Introduction

A prolonged ED length of stay (LOS) keeps doctors and nurses longer occupied with one patient, and decreases the effective capacity which contributes to overcrowding. Emergency department (ED) overcrowding is associated with worse patient outcomes and satisfaction among health workers and patients [1–3]. Crowding is also a problem in the Netherlands; 68% of the ED managers experienced crowding several times a week or even daily [4]. In the UK the 4-h rule was introduced to restrict the ED work-up time [5]. Also in our hospital in the Netherlands crowding is an issue affecting satisfaction of patients and medical personal. Therefore, an ED LOS of 4 h was adopted as an important cut-off point to indicate prolonged LOS.

Reduction of ED LOS may contribute to reduction of ED overcrowding [2, 6]. Despite the fact that numerous ED and patient characteristics have been associated with prolonged ED LOS, a recent systematic review demonstrated that previous studies were inappropriate to decide which factors need to be targeted to optimize ED logistics [7]. Several issues arise in these studies. First, almost all have been retrospective studies while real-time measurements are especially important when studying time delays in ED logistics. Secondly, often only a few factors have been measured while all potential bottlenecks should be taken into account to understand which factors should be targeted and can actually be modified in clinical practice. For example, age has frequently been associated with ED LOS [7–9]. However, this could be caused by age-related differences in comorbidity and disease severity. Comorbidity has been associated with a higher number of ED visits, hospital admissions, readmissions and health care costs [10–13]. Comorbidity increases, in combination with multiple presenting complaints, the complexity of care and could contribute to a prolonged ED LOS. Typically, patients of a tertiary care centre have more comorbidities and presenting complaints.

Finally, previous studies mostly originate from the USA or Canada [7]. The European ED setting is quite different with regard to the number of ED visits and ED LOS. In addition, EDs are staffed by both ED physicians and other specialists (as opposed to only ED physicians), and the general practitioner has an important role as gate keeper in referral of patients to the hospital [14].

### Importance

Successful solutions for reduction of ED LOS in the European health-care setting can only be developed if ED patient flows are prospectively studied and independent modifiable ‘bottlenecks’ are identified by using multivariable prediction modelling [15].

### Aim of the study

The purpose of the present study was therefore two-fold: first, to identify independent determinants of prolonged ED LOS (including all important patient, doctor and ED (management) factors). Secondly, to measure (in real time) the absolute time delays associated with the modifiable independent determinants of prolonged ED LOS, and the delays associated with decision-making and ED discharge.

## Methods

### Study design and setting

This was a prospective observational cohort study including ED patients of the Leiden University Medical Centre (LUMC), a Dutch tertiary care centre with ~ 26.000 ED visits each year. Data were collected from 16 December 2014 to 11 February 2015.

In the Netherlands approximately 84 hospitals have EDs, the highest concentration in the western part of the Netherlands. Patients are either referred to the ED by a general practitioner (GP) or are self-referred (which is accepted). Furthermore, the Netherlands is divided in regional ambulance services. In the region of the LUMC the ambulance service “Hollands Midden” is responsible for most of the ambulance transports. This district covers 875 km2 with 760.000 citizens and is accountable for ~ 60,000 transports per year. In 95% of the rides ambulances arrive at the scene within 15 min of the dispatch call. The Dutch ED setting is characterized by the presence of both ED physicians and other specialists in the ED. The ED is staffed 24/7 by ED physicians, who are responsible for self-referred patients, trauma and critical care and patients who are directly referred to an ED physician.

The study was approved by the medical ethics committee of the LUMC, who waived the need for individual informed consent because of the purely observational character of the study (Protocol number P14.288).

### Participants

All consecutive patients presenting to the ED between 10 a.m. and 10 p.m. were included during 30 randomly chosen days, including weekends, in a 9 week time period [16]. Approximately 70% of all ED patients per 24 h arrive in the selected time period_._ It is important to note that this way of sampling does not create selection bias [17].

### Data collection and measurements

**Fig. 1:**
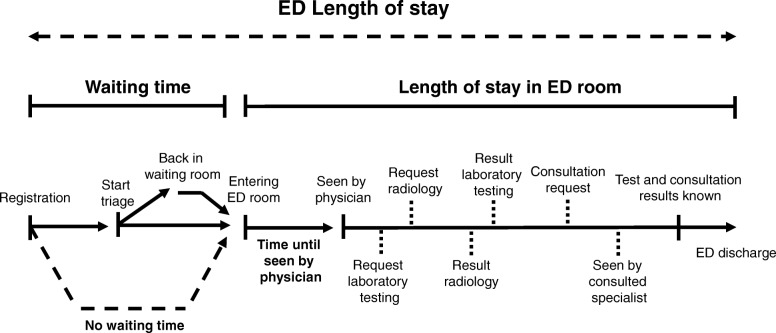
Time components of emergency department (ED) length of stay (LOS). Times have been prospectively registered. In case of no waiting time, the time of entering the ED room was equal to the time of registration

In Fig. 1 the patient flow through the ED is depicted schematically.

Demographic variables, referral status, type of arrival (by ambulance or own transport), triage category, treating specialty, comorbidity, type and number of presenting complaints, diagnostic testing, consultations of other specialties in the ED, number of patients present in the ED at the time of ED registration and final disposition, were prospectively registered by an observer (DvdV) in a standardized case report file in SPSS (SPSS V.23.0, IBM, New York, USA). Transit times (ED bed placement time, ED departure time and time of physician arrival, consultation request and arrival of the consulted physician, time of physically leaving the ED) were measured in real-time.

In the digital hospital information system (Chipsoft, Amsterdam) information about the ED registration time, the day of discharge and times of diagnostic testing (times when laboratory analysis was started or radiology imaging was requested and times when these were finished) were obtained. In the final conclusion of the medical file the total number of presenting complaints or problems were quantified. The triage category and triage complaint were registered according to the Manchester Triage System (MTS).

Comorbidity was assessed in two ways: the Charlson comorbidity index (CCI) was calculated and comorbidities were registered by an organ-based method [18]. For exact definitions and scoring system for the comorbidities see online Additional file 1.

ED LOS was calculated by subtracting the ED registration time from the time that the patient physically left the ED. If the ED departure time was after 10 pm, the time registered in the digital hospital information system was used. Waiting time was calculated by subtracting the ED registration time from the time a patient was placed in an ED treatment room. Time until seen by a physician was calculated by subtracting ED room entrance time from the time the physician had arrived.

### Outcome measure

The primary outcome measure of the present study was a total ED LOS of more than 4 h.

### Data analysis

#### Sample size estimation

For the multivariable logistic regression analysis we used the rule of thumb that approximately 10 events per covariate were needed to prevent overfitting. Because we wanted to put 27 variables in the model, 270 events were needed.

#### Descriptive statistics

Continuous data were presented as mean (standard deviation: SD) if normally distributed and median (interquartile range: IQR) if data were rightly skewed. Categorical data were presented as number (%). Differences between continuous data were analyzed with student t-tests or Mann-Whitney U-tests as appropriate. Furthermore, chi-square tests were used for analyzing descriptive categorical data.

#### Main statistical analysis

Multivariable binary logistic regression analysis with backward entry of arrival type (self-referral or by ambulance), age, triage category, treating specialty, diagnostic testing, consultations, disposition (hospital admission or discharge home), number of comorbidities, types and number of presenting complaints and number of patients present in the ED at the time of ED registration, was used to identify the independent determinants of ED LOS longer than 4 h. Before we entered variables in the model we examined if the variable had a linear relation with the outcome, if not, we categorized the variable and created dummy variables.

We calculated ED LOS for the total cohort and for patients with diagnostic testing or consultations. The Hosmer-Lemeshow test was used to assess goodness of fit. The c-statistic was used as a measure of discriminative performance of the prediction model. Variance influence factors (VIF) were assessed to assess if multicollinearity is a problem. Multicollinearity was not considered as a problem if the VIF was below 3.

The odds ratios (ORs) with 95%-confidence intervals (CI) were reported. *P*-values < 0.05 were considered significant. All data were analyzed using SPSS statistics 23.0.0 software (IBM, New York, USA).

## Results

### Patient inclusion and characteristics

**Fig. 2:**
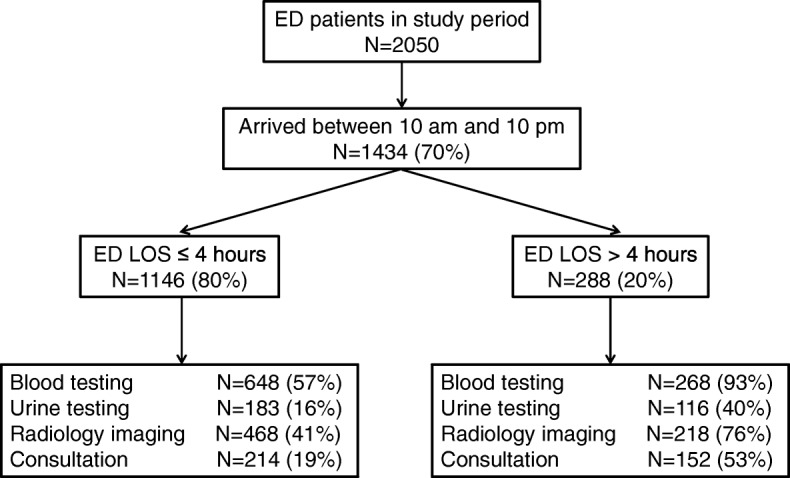
Patient inclusion and flow through study

During the inclusion period a total of 2050 patients visited the ED, of which 1434 patients (70%) arrived between 10 am en 10 pm. The patient flow through the study is visualized in Fig. 2. Of the included patients, 1146 patients (80%) had an ED LOS < 4 h and 288 patients (20%) had an ED LOS > 4 h. Patient characteristics are summarized in Table 1. Patients with an ED LOS > 4 h were on average older (58 (21) versus 45 (26)) and had more comorbidities (2 (1–3) versus 1 (0–2). In addition, the number of blood testing (93% versus 57%), urine testing (40% versus 16%), radiology imaging (76% versus 41%), consultations (53% versus 19%) and admissions to a ward (51% versus 19%) was higher in patients with an ED LOS > 4 compared to patients with an ED LOS < 4 h.

**Table 1:** Patient and logistical factors as a function of emergency department length of stay

	Total population	ED LOS ≤ 4 h	ED LOS > 4 h
N (%)	1434	1146 (80)	288 (20)
Demographics			
Age, mean (SD)	48 (26)	45 (26)	58 (21)
Gender (male, %)	749 (52)	599 (52)	150 (52)
Referral status (%)			
Self-referral	469 (33)	400 (35)	69 (24)
General practitioner	612 (43)	448 (39)	164 (57)
Specialist	353 (25)	298 (26)	55 (19)
Arrival by ambulance (n, %)	484 (34)	364 (32)	120 (42)
Number of presenting complaints, median (IQR)	1 (1–1)	1 (1–1)	1 (1–2)
Triage category ^a^ (n, %) (4)			
Red	32 (2)	32 (3)	0 (0)
Orange	371 (26)	279 (24)	92 (32)
Yellow	682 (48)	518 (45)	164 (57)
Green	337 (24)	305 (27)	32 (11)
Blue	8 (1)	8 (1)	0 (0)
Number of co-morbidities, median (IQR)	1 (0–2)	1 (0–2)	2 (1–3)
CCI, median (IQR)	0 (0–0)	0 (0–1)	1 (0–2)
Triage complaint (n,%)			
Headache	16 (1)	8 (1)	8 (3)
Dyspnoea	144 (10)	103 (9)	41 (14)
Chest pain	114 (8)	74 (7)	40 (14)
Palpitations	41 (3)	24 (3)	7 (2)
Abdominal pain	135 (9)	98 (9)	37 (13)
Malaise	274 (19)	200 (18)	74 (26)
Small traumatic injury	323 (23)	299 (26)	24 (8)
Syncope	43 (3)	36 (3)	7 (2)
Other	344 (24)	294 (26)	50 (17)
Treating physician (n, %)			
ED physician	715 (50)	608 (53)	107 (37)
Internal medicine	221 (15)	141 (12)	80 (28)
Cardiology	117 (8)	85 (7)	32 (11)
Surgery	71 (5)	58 (5)	13 (5)
Neurology	78 (5)	52 (5)	26 (9)
Other specialties	232 (16)	202 (18)	30 (10)
Diagnostic tests (n, %)			
Blood testing	916 (64)	648 (57)	268 (93)
Urine testing	299 (21)	183 (16)	116 (40)
Radiology imaging	686 (48)	468 (41)	218 (76)
Consultation of other specialty in ED (n, %)	366 (26)	214 (19)	152 (53)
Number of patients in ED at the time of ED registration, mean (SD)	11 (5)	11 (5)	11 (5)
Disposition (n, %) (1)			
Home	916 (64)	785 (69)	131 (46)
Admission ward	46 (32)	317 (28)	148 (51)
Admission ICU/MCU	46 (3)	37 (3)	9 (3)
Other^b^	6 (1)	6 (3)	0 (0)
ED LOS (min), median (IQR)	156 (98–225)	134 (87–180)	301 (270–357)

Patient characteristics are presented for the total population, patients who had an ED LOS ≤ and > 4 h. Continuous data are presented as mean (SD) or median (IQR) and categorical data as number (n),%). The number of missing cases are denoted between brackets for each variable

^a^ The presented ‘triage category’ and ‘triage complaint’ were according to the Manchester Triage System (MTS)

^b^ Of the 6 patients with another final disposition, 5 patients died on the ED and 1 patients returned to the psychiatric hospital were the patient was admitted before arrival at the ED

Abbreviations: *CCI* Charlson Comorbidity Index, *ED* Emergency Department, *LOS* Length of Stay, *IC* Intensive Care Unit, *MCU* Medium Care Unit

### Independent determinants of ED LOS > 4 h

**Fig. 3:**
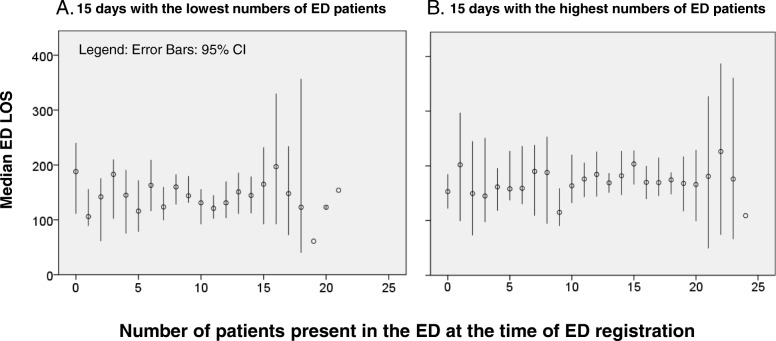
Emergency department (ED) length of stay (LOS) as a function of the number of patients present in the ED at the time of ED registration in the 15 days with the lowest number of patients (**a**) and the 15 days with the highest number of patients per day (**b**)

In Table 2, the independent determinants for ED LOS > 4 h are shown. The adjusted ORs (95%-CI) were 3.45 (1.95–6.11) for blood testing, 1.79 (1.21–2.63) for urine testing, 3.02 (2.13–4.30) for radiology imaging, 5.92 (4.08–8.60) for consultations in the ED, 2.10 (1.21–3.66) for internal medicine and 2.24 (1.06–4.73) for neurology as treating physician, and 3.66 (1.08–12.41) for headache and 2.91 (1.49–5.71) for chestpain as presenting complaint. Age, number of comorbidities, and admission to a ward were not an independent determinant of prolonged ED LOS. Patients who were admitted to an ICU or MCU had a much shorter ED LOS; adjusted OR of 0.26 (0.10–0.66). The number of patients present in the ED at the time of ED registration was merely a determinant if 15 patients or more were present in the ED at the time of ED registration; Adjusted OR of 2.21 (1.30–3.75). In Fig. 3, ED LOS is depicted as a function of number of patients present in the ED at the time of ED registration in the 15 days with the lowest number of patients (3A) and the 15 days with the highest number per day (3B). Even during the busiest days ED LOS barely increased with increasing number of patients present in the ED.

**Table 2:** Uni- and multi-variable binary logistic regression analysis to predict ED lengths of stay > 4 h

	Univariable analysis OR (95%-CI)	Multivariable analysis Adjusted OR (95% CI)
Age	1.02 (1.02–1.03)	–
Gender (male)	0.99 (0.77–1.29)	–
Arrival by ambulance	1.54 (1.18–2.00)	–
Number of presenting complaints	1.63 (1.42–1.88)	1.19 (1.00–1.41)
Referral status		
Self-referral	Ref	
General practitioner	2.12 (1.55–2.90)	–
Specialist	1.07 (0.73–1.57)	–
Triage category		
Green and blue	Ref	
Yellow	3.10 (2.07–4.64)	–
Red and orange	2.89 (1.88–4.46)	–
Number of comorbidities^a^	1.52 (1.37–1.68)	–
Triage complaint		
Other	Ref	Ref
Headache	5.88 (2.11–16.39)	3.66 (1.08–12.41)
Dyspnoea	2.34 (1.46–3.75)	1.48 (0.83–2.65)
Chest pain	3.18 (1.95–5.18)	2.91 (1.49–5.71)
Palpitations	1.21 (0.51–2.88)	0.69 (0.24–2.00)
Abdominal pain	2.22 (1.37–3.60)	1.20 (0.65–2.22)
Malaise	2.18 (1.46–3.25)	1.37 (0.84–2.24)
Small traumatic injury	0.47 (0.28–0.79)	0.91 (0.49–1.70)
Syncope	1.14 (0.48–2.71)	1.03 (0.39–2.77)
Treating physician		
Other specialties^b^	Ref	Ref
ED physician	1.19 (0.77–1.83)	0.67 (0.38–1.17)
Internal medicine	3.82 (2.38–6.12)	2.10 (1.21–3.66)
Cardiology	2.54 (1.45–4.43)	1.36 (0.63–2.89)
Surgery	1.51 (0.74–3.08)	1.92 (0.80–4.65)
Neurology	3.37 (1.83–6.18)	2.24 (1.06–4.73)
Diagnostic testing		
Blood testing	10.30 (6.44–16.46)	3.45 (1.95–6.11)
Urine testing	3.55 (2.67–4.71)	1.79 (1.21–2.63)
Radiology imaging	4.51 (3.36–6.05)	3.02 (2.13–4.30)
Consultation	4.87 (3.70–6.41)	5.92 (4.08–8.60)
Number of patients in ED at the time of ED registration		
0–5 patients	Ref	Ref
6–10 patients	1.23 (0.81–1.86)	1.22 (0.76–2.00)
11–15 patients	1.30 (0.87–1.96)	1.37 (0.85–2.23)
> 15 patients	1.65 (1.05–2.58)	2.21 (1.30–3.75)
Disposition		
Home	Ref	Ref
Admission ward	2.80 (2.14–3.67)	2.00 (0.75–1.40)
Admission ICU/MCU	1.63 (0.79–3.36)	0.26 (0.10–0.66)

Uni- and Multi-variable binary logistic regression analysis was performed with backward entry of all variables. Data are presented as odds ratio (OR (95% CI)). The Hosmer-Lemeshows test had a *p*-value of 0.905. The area under the curve (c-statistic) was 0.850 (0.827 to 0.873). The VIFs varied between 1.00 and 1.47, never above 3. *N* = 1434

A “– “indicates that the variable was eliminated from the model, and no independent determinant in the multivariable regression analysis

^a^ The CCI and number of comorbidities were analyzed separately. Both were not associated with ED LOS > 4 h

^b^ Other specialties were pediatrics, ophthalmology, dermatology, otorhinolaryngology, psychiatry and gynecology

Abbreviations: *ED* emergency department, *Ref* reference, *OR* odds ratio, *CI* confidence interval

### Time components of ED LOS

The several time components, which contribute to the total ED LOS, are illustrated in Fig. 1.

The absolute time delays of diagnostic testing and consultations are shown in Table 3. In the total patient cohort the median ED LOS was 156 min (98–225). In 1123 patients (78%) at least one consultation or diagnostic test was requested, which increased the total ED LOS to 177 min (129–242). The median time between patient registration and requesting diagnostic testing or consultations ranged between 23 (13%) to 82 (46%) minutes and the median time between requesting and having the results ranged between 26 (15%) to 69 (39%) minutes of the total ED LOS. The median time between finishing diagnostic testing or consultation and ED discharge was 48 (27%) minutes of the total ED LOS.

**Table 3:** Time components of total ED length of stay

	Number (%)	Median time (IQR)	ED LOS (IQR)
All included patients	1434 (100)	–	156 (98–225)
Waiting time (min) (2)	–	10 (3–27)	–
Time until seen by physician (min) (410)	–	9 (1–29)	–
Blood testing	916 (64)	–	191 (139–256)
Time between patient registration and start analysing blood (min)	–	23 (14–40)	–
Time between start blood analysing and test result is registered in HIS (min)	–	69 (53–101)	–
Urine testing	299 (21)	–	217 (163–291)
Time between patient registration and start analysing urine (min) (123)	–	82 (34–120)	–
Time between start urine analysing and test result is registered in HIS (min) (123)	–	29 (18–53)	–
Radiology imaging	686 (48)	–	195 (136–270)
Time between patient registration and radiology request (min)	–	39 (17–82)	–
Time between radiology request and test result registered in HIS (min)	–	37 (20–65)	–
Consultation	366 (26)	–	222 (155–294)
Time between patient registration and consultation request (min) (113)		70 (39–106)	–
Time between consultation request and examination of the patient by the consulted specialist (min) (191)		26 (11–47)	–
Diagnostic testing or consultation	1123 (78)	–	177 (129–242)
Time between finishing last diagnostic testing or consultation and ED discharge (min) (23)	–	48 (20–88)	

Time components of total ED length of stay are presented as median (IQR) and categorical data are presented as frequency (%). The ED LOS of each subgroup of the total population is shown

In total, 796 (56%) patients had diagnostic tests of which 193 (24%) patients had advanced triage, known as starting diagnostic testing before entering the ED room. In 116 patients, the median time between starting of the blood analysis and entering ED room was 23 min (IQR 9–54). Likewise, urine analysis started in 8 patients and radiology imaging started in 78 patients before entering ED room. The median times and IQR were respectively, 12 min (2–22) and 20 min (11–40). The urine analysis was not finished in 9 patients before admission and the blood analysis was not finished in 68 patients before admission. In these patients the times between finishing diagnostic testing and ED discharge were negative

Abbreviations: *ED* Emergency Department, *LOS* Length of Stay, *HIS* Hospital Information System

## Discussion

The main conclusion of this study is that laboratory/radiology testing and consultations are necessary in 78% of all ED patients and are the most important independent determinants of prolonged ED LOS. More importantly, between 42 and 66% of the total ED LOS is spent on laboratory/radiology testing and consultations, and the decision-making and discharge process thereafter.

In a recent systematic review it has been suggested that despite many studies investigating factors associated with ED LOS, accurate conclusions cannot be drawn about how to reduce ED LOS. Reasons are that studies were mostly retrospective and some important factors like specific presenting complaints or co-morbidities were not assessed, nor were multivariable prediction models developed to correct for potential confounding [7]. In the present study we therefore aimed to prospectively investigate all important ED and patient factors affecting ED LOS. This yielded some important insight in factors affecting ED logistics. Firstly, in contrast to previous studies age *per se* was not independently associated with prolonged ED LOS [7–9]. Our study suggests that this is probably explained by a higher percentage of diagnostic testing and consultations in older people [16].

Secondly, in previous studies the association between comorbidity and ED LOS has been insufficiently examined [7]. In addition, the large variability in comorbidity scoring methods makes it difficult to compare our findings with previous literature [19]. In an attempt to increase the comparability, we examined the influence of comorbidity in two ways: the number of comorbidities and the CCI were analysed separately. Neither were independent determinants for prolonged ED LOS, probably because the larger number of diagnostic tests and/or consultations results in longer ED LOS in patients with more comorbidity, rather than the number of comorbidities per se.

Thirdly, in our study an association between arrival by ambulance and ED LOS was not found, possibly because arrival by ambulance is a measure of disease severity and/or complexity which has been quantified in our study by triage category, number of comorbidities, and number of presenting complaints and problems. The variable “arrival by ambulance” is probably eliminated from the multivariable regression model because the larger number of diagnostic tests and consultations associated with these other measures of disease severity and/or complexity are the independent determinants of prolonged ED LOS.

Finally, although in many studies hospital admission was associated with prolonged ED LOS [7], our study found the opposite. One reason is that we discriminated between hospital admission to a normal ward or to an MCU/ICU. Admission to a normal ward was not associated with prolonged ED LOS and ICU admission was even associated with a short ED LOS, probably because patients who need ICU admission are often taken care of with a team with multiple expertises in the shockroom. During this team approach, diagnostic testing and consultations are accelerated and done simultaneously because of the acuity.

Hospitalized patients are more ill and complex, which could explain the association with prolonged ED LOS in previous studies. However, in our study, this complexity is reflected by the variables “number of presenting complaints and co-morbidities” and not by the variable “hospital admission,” explaining the lack of an association between hospital admission and prolonged ED LOS in our study. Thus, it is not the hospitalization per se that results in prolonged ED LOS but the associated complexity and diagnostic tests and consultations.

Our study has several implications for clinical practice and offers suggestions for improvements. The time delays caused by waiting time and time until seen by physician are short, which implicates that time-saving could mainly be achieved by reducing treatment time.

First, our study suggests that advanced triage, i.e. early initiation of diagnostic testing at the time of triage [20] would reduce ED LOS. In our study, merely 24% of the patients with diagnostic testing were selected for advanced triage. Moreover, reducing the number of additional blood analyses could improve the time between requesting diagnostic tests and their result. For example, erythrocyte sedimentation rate and C-reactive protein may not be useful for clinical decision making in the ED [21]. Troponins often need to be assessed multiple times.

Secondly, in patients with multiple comorbidities, delays caused by diagnostic testing and consultations could be prevented if they are immediately hospitalized once a clear indication for hospital admission exists, i.e. need for supplemental oxygen or intravenous medication. Awaiting test and consultations results in a clinical decision unit would be a suitable option for these patients, provided that patient safety is not jeopardized. In patients who do not need hospitalization but who do require outpatient follow-up some diagnostic tests and consultations might be done in the outpatient setting.

Thirdly, reduction of ED LOS could be achieved by clear agreements on who admits patients with multiple comorbidities and presenting complaints, and by increasing the presence of staff members in the ED, as has been suggested in a recent study [22]. This is expected to reduce ED LOS because in the Netherlands referred patients are mostly seen by residents of the treating specialty who, before making decisions, need to discuss patients with their supervising staff members who are often not present in the ED because of obligations elsewhere, i.e. in the operation theatre, outpatient department or ward.

Finally, if hospital admission is indicated, ED physicians have to consult the resident of the admitting specialty. If instead hospital admission could directly be discussed with the consultant of the admitting specialty a further reduction of ED LOS would be possible since consultations are associated with a large time delay.

### Limitations

Although our study has several strengths like the prospective design (with real time measurements of time delays) our study has also some limitations. Firstly, ED crowding was not measured according to a validated crowding score because existing crowding scores are not validated in the Dutch ED setting. However, the number of patients present in the ED was measured in real time and is a major factor related to overcrowding [23].

Secondly, our results may not be applicable for all hospitals because tertiary care centres usually treat more complex patients. As a result, the impact of consultations on ED LOS could be overestimated in urban hospitals, because of a higher consultation rate in tertiary care centres [24]. Thirdly, although our sampling method should not have introduced selection bias [17], it is theoretically possible that during night hours, laboratory and radiology services are more short-staffed, probably delaying diagnostic testing results. In addition, staff members are less available for supervision. Therefore, the impact of consultations and diagnostic testing on ED LOS could have been underestimated.

Finally, although in our hospital the number of patients per month is fairly stable throughout the year, there could be some impact of season. However, given the low impact of number of patients present in the ED on ED LOS (Fig. 3), it is not likely that variation in number of ED presentation with season does affect the independent determinants of prolonged ED LOS. It is possible that the type of patients varies with season but we adjust for that in the multi-variable regression analysis.

## Conclusions

Reduction of ED LOS is possible by optimizing the process of laboratory/radiology testing and consultations and facilitation of the decision-making and discharge procedures. Future studies should investigate if an accelerated hospital admission protocol will reduce ED LOS.

## Additional file

Additional file 1: Comorbidity scoring system. (DOC 32 kb)

## Abbreviations

AUC: Area under the curve
AVPU: Alert verbal pain unresponsive
CCI: Charlson comorbidity index
CI: Confidence interval
DNR: Do not resuscitate
ED: Emergency department
H: Hour
ICU: Intensive care unit
IQR: Inter quartile range
LOS: Lengths of stay
LUMC: Leiden University Medical Centre
MCU: Medium Care unit
Min: Minute
MTS: Manchester Triage System
OR: Odds ratio
SD: Standard deviation
SEM: Standard Error of the Mean
USA: United States of America
VIF: Variance inflation factor
