# Supplementary material for: Independent determinants of prolonged emergency department length of stay in a tertiary care centre: a prospective cohort study
Source: Scand J Trauma Resusc Emerg Med. 2018 Sep 20;26:81. doi: 10.1186/s13049-018-0547-5 (PMC6148782; doi:10.1186/s13049-018-0547-5)
Supplement: Supplementary file 1 — Comorbidity scoring system. (DOC 32 kb) [file 13049_2018_547_MOESM1_ESM.doc]

| **Additional file 1. Comorbidity scoring system** | |
| --- | --- |
| **Group of diseases** | **Included diseases** |
| Chronic pulmonary disease | Lung fibrosis, COPD, asthma, transplantation, etcetera |
| Cardiovascular disease | Myocardial infarction, heart failure, coronary heart disease, arrhythmia, heart valve disease, cardiomyopathy, hypertension, etcetera |
| Chronic renal disease | IgA nephropathy, hypertensive nephropathy en diabetic nephropathy, granulomatosis with polyangiitis, systemic lupus erythematosus (if kidneys are involved), transplantation, etcetera |
| Chronic liver and gastrointestinal disease | Cirrhosis, chronic hepatitis and portal hypertension, inflammatory bowel disease, primary sclerosing cholangitis, chronic pancreatitis, ulcer disease, etcetera |
| Autoimmune and rheumatologic diseases | Diabetes mellitus, rheumatoid arthritis, systemic lupus erythematosus, thyroid diseases, etcetera |
| Solid malignancy without metastases | Solid malignancy without metastases |
| Metastatic cancer and hematologic malignancies | Haemophilia, thalassemia, hemochromatosis, etcetera |
| Chronic neurological disease | Multiple sclerosis, Parkinson's disease, dementia, epilepsy, transient ischemic attack, cerebro vasculair accident, mental retardation, etcetera |
| Chronic psychiatric disorder | Anxiety, depression, post-traumatic stress disorder, personality disorders, psychosis, etcetera |
| Chronic dermatologic diseases | Eczema, psoriasis, etcetera (which require medication) |
